# The Real-World Efficacy and Side Effects of Different Nivolumab Regimens in Japanese Patients with Advanced Melanoma: A Single-Center Retrospective Study

**DOI:** 10.3390/cancers17142299

**Published:** 2025-07-10

**Authors:** Ken Horisaki, Shusuke Yoshikawa, Wataru Omata, Arata Tsutsumida, Yoshio Kiyohara

**Affiliations:** 1 Department of Dermatology, Shizuoka Cancer Center, Shizuoka 411-8777, Japan; k.horisaki@scchr.jp (K.H.); w.omata@scchr.jp (W.O.); a.tsutsumida@scchr.jp (A.T.); y.kiyohara@scchr.jp (Y.K.); 2 Department of Dermatology, Nagoya University Graduate School of Medicine, Nagoya 466-8560, Japan

**Keywords:** melanoma, nivolumab, 240 mg, 480 mg, retrospective cohort, immune-related adverse event

## Abstract

**Background/Objectives**: Nivolumab is a key therapy for advanced-stage melanoma; however, limited data are available from Asian populations comparing the efficacy and side effects of four dosing regimens: 3 mg/kg every 2 weeks (3mg/kgQ2W), 2 mg/kg every 3 weeks (2mg/kgQ3W), 240 mg every 2 weeks (240mgQ2W), and 480 mg every 4 weeks (480mgQ4W). This retrospective study evaluated Japanese patients with advanced melanoma treated with various nivolumab regimens to assess the impact of dosing interval and dosage on treatment efficacy and immune-related adverse events (irAEs). **Methods**: We reviewed the records of 153 participants with stage IV melanoma who received nivolumab monotherapy between February 2012 and December 2024 at Shizuoka Cancer Center. Patients were categorized by nivolumab regimen, dosing interval, and dose per body weight. We then compared treatment efficacy and incidence of irAEs across groups. **Results**: No significant differences were observed in objective response rate (ORR), progression-free survival (PFS), overall survival (OS), or irAE incidence between the 240mgQ2W and 480mgQ4W groups. Similar results were observed in the 3mg/kgQ2W and 2mg/kgQ3W groups. However, participants who received nivolumab within 3 weeks exhibited a significantly higher ORR than those who received nivolumab more than 3 weeks. No significant differences were found in PFS or OS. **Conclusions**: The administration of nivolumab at shorter intervals may provide short-term benefits in Japanese patients with advanced melanoma. However, long-term efficacy and side effects did not differ significantly across the studied nivolumab regimens.

## Simple Summary

Nivolumab is currently administered for advanced melanoma at either 240 mg every 2 weeks or 480 mg every 4 weeks. However, limited data are available comparing the effectiveness of these regimens. In this retrospective study, we analyzed 153 Japanese patients with melanoma, including those who previously received 3 mg/kg every 2 weeks or 2 mg/kg every 3 weeks. The analysis indicated that shorter administration intervals may improve the objective response rate, although long-term efficacy and side effects did not differ significantly by regimen or dosage. Given the comparable efficacy and toxicity between the 240 mg and 480 mg regimens, we recommend that clinicians discuss options with patients and select the most suitable regimen collaboratively.

## 1. Introduction

The advent of immune checkpoint inhibitors (ICIs) has significantly transformed systemic therapy for malignant melanoma (MM) [1]. Nivolumab, a human monoclonal antibody targeting programmed cell death protein 1 (PD-1), is a representative ICI and is effective in cancer immunotherapy due to its unique immunomodulatory properties. The PD-1 pathway has been shown to play an important role in immune regulation. PD-1, expressed on activated T cells, acts as a key checkpoint molecule that downregulates T cell responses through multiple mechanisms. Nivolumab specifically binds to the PD-1 receptor and inhibits its interaction with both PD-L1 and PD-L2, thereby releasing the suppression of immune responses via the PD-1 pathway and exerting its antitumor therapeutic effect [2]. Nivolumab was initially approved as a weight-based dose of 3 mg/kg every 2 weeks (3mg/kgQ2W) and 2 mg/kg every 3 weeks (2mg/kgQ3W) for the treatment of MM. In 2017, fixed-dose regimens of 240 mg every 2 weeks (240mgQ2W) and 480 mg every 4 weeks (480mgQ4W) were approved and are currently in clinical use.

Clinical trials have reported response rates of approximately 40–50% for nivolumab monotherapy in MM [3,4,5]; however, more than half of patients with MM do not derive benefit. Moreover, the risk of immune-related adverse events (irAEs) remains a critical consideration. ICIs disrupt the immune balance in the body, reducing T cell tolerance and resulting in a series of irAEs that have different symptoms, durations, and severities to the toxicities of traditional oncology drugs [6]. Among patients with MM treated with nivolumab monotherapy, irAEs occur in approximately 60–80% of cases [3,7,8], with severe irAEs observed in roughly 10–20% [3,5,7,8,9]. In the combination therapy with nivolumab and ipilimumab, the incidence of any-grade irAEs was reported to be approximately 80–90%, and severe irAEs was reported to be approximately 50% [3,4,5,10,11]. Therefore, although the incidence of irAEs with nivolumab monotherapy is less than that with the combination therapy, it is still a significant issue. Consequently, there is a pressing need to optimize nivolumab administration to improve efficacy while minimizing adverse effects. Further, studies indicate that Asian populations exhibit a higher incidence of acral and mucosal MM subtypes and demonstrate lower response rates to ICIs compared with Western populations [12,13,14]. Regarding nivolumab monotherapy, the response rate to nivolumab in advanced MM has been reported to be less than 30% in Japanese patients compared with more than 40% in Westerners [3,4,12,14]. These findings underscore the necessity of evaluating ICI efficacy across different racial groups.

Previous investigations comparing weight-based and fixed-dose regimens found no significant differences in efficacy or toxicity [15,16,17]. While cohort studies have explored adverse effects between the 240mgQ2W and 480mgQ4W regimens in Western populations [18,19,20], real-world data specific to Asian populations remain limited.

To address this gap, the present study retrospectively analyzed Japanese patients with advanced MM treated with various nivolumab regimens, including fixed-dose options. We aimed to compare the efficacy and safety of different dosing intervals and weight-adjusted doses, ultimately identifying the optimal regimen for patients of Asian descent.

## 2. Materials and Methods

### 2.1. Study Population and Data Collection

This retrospective cohort study was conducted at Shizuoka Cancer Center, Japan. We reviewed the records of patients with stage IV MM who received nivolumab monotherapy between February 2012 and December 2024. The inclusion criteria included the following: (i) pathologically confirmed MM; (ii) primary sites including all skin, mucosa, uvea, and unknown primary sites; (iii) classified as stage IV according to the American Joint Committee on Cancer (AJCC) 8th edition classification [21]; and (iv) received one of four nivolumab regimens (3mg/kgQ2W, 2mg/kgQ3W, 240mgQ2W, and 480mgQ4W). If patients transitioned to a different regimen during treatment, only the initial regimen was considered for analysis. For AJCC staging, the cutaneous MM criteria were applied for unclassifiable genital, anal, or urinary tract tumors. Patients who had received prior pembrolizumab monotherapy were excluded.

Collected clinical data included participants’ age, sex, Eastern Cooperative Oncology Group Performance Status (ECOG-PS), body weight, primary tumor location, prior surgical and systemic treatments, stage IV classification, B-rapidly accelerated fibrosarcoma (BRAF) mutation status, irAEs, and lactate dehydrogenase (LDH) levels at nivolumab initiation.

### 2.2. Efficacy Assessment

The primary outcomes included the objective response rate (ORR), disease control rate (DCR), progression-free survival (PFS), and overall survival (OS), stratified by nivolumab regimen, dosing interval, and dose per body weight. The secondary outcome was the incidence of irAEs. Treatment response was evaluated using the Response Evaluation Criteria in Solid Tumors (RECIST) version 1.1 [22]. The ORR was defined as the proportion of participants achieving complete response (CR) or partial response (PR). The DCR was defined as the proportion of patients achieving either a CR, PR, or stable disease (SD). The objective response was assessed every 2–3 months from the start of nivolumab treatment. PFS and OS were defined from the time nivolumab treatment was initiated until radiological or clinical tumor progression (PFS), death from any cause (OS), or final follow-up (PFS and OS). Finally, irAEs were graded according to the Common Terminology Criteria for Adverse Events (CTCAE) version 5.0 [23].

### 2.3. Ethics Statement

This retrospective cohort study was approved by the Institutional Review Board of Shizuoka Cancer Center (approval number: J2025–50). The need for informed consent was waived due to this study’s retrospective observational design. All personal data were managed in accordance with the ethical standards of the 1964 Declaration of Helsinki and its subsequent amendments.

### 2.4. Statistical Analysis

Baseline characteristics were compared using the Mann–Whitney U test for continuous variables and the chi-squared or Fisher’s exact tests for categorical variables. These tests were also used to assess the ORR and irAE frequencies. Receiver operating characteristic (ROC) curve analysis determined the optimal cutoff value for nivolumab dose per body weight. Dosing interval grouping was based on a cutoff value between 2 and 4 weeks that maximized equal numbers in both groups. PFS and OS were estimated using the Kaplan–Meier method. Differences in PFS and OS among treatment groups were analyzed using log-rank tests. Cox regression analysis was performed to calculate hazard ratios (HRs) for covariates affecting PFS and OS. *p*-values less than 0.05 were considered statistically significant. All statistical analyses were performed using EZR ver1.68 (Saitama Medical Center, Jichi Medical University, Saitama, Japan), a graphical interface for R (The R Foundation for Statistical Computing, Vienna, Austria).

## 3. Results

### 3.1. Baseline Characteristics

The clinical characteristics of 153 patients with stage IV MM by nivolumab regimen are summarized in Table 1. Forty (53.3%) participants in the 240mgQ2W group and thirty-five (46.7%) in the 480mgQ4W group showed no clinically significant differences. In contrast, forty-one (52.6%) participants in the 2mg/kgQ3W group and thirty-seven (47.4%) in the 3mg/kgQ2W group exhibited differences in BRAF gene test results, although no other significant differences were identified.

Seventy-seven (50.3%) participants received nivolumab at dosing intervals of ≥3 weeks (Q ≥ 3W), while 76 (49.7%) were treated at intervals of <3 weeks (Q < 3W) (Table 2). Apart from BRAF gene test results, no significant differences were observed between these groups. The ROC curve indicated that the optimal cutoff value for nivolumab dose per body weight was 4.6 mg/kg. The area under the curve was 0.616, the sensitivity was 0.712, and the specificity was 0.556. Based on this cutoff, 101 (66.0%) participants were categorized into the <4.6 mg/kg group and 52 (34.0%) were categorized into the ≥4.6 mg/kg group. First-line nivolumab therapy was more common in the <4.6 mg/kg group than the ≥4.6 mg/kg group (83.2% vs. 53.8%, respectively; *p* < 0.001). The overall median follow-up time was 10.6 months, with no significant differences in follow-up time between any of the groups.

### 3.2. Objective Response

The ORR tended to be higher in the 240mgQ2W group than in the 480mgQ4W group (*p* = 0.082), and in the 3mg/kgQ2W group compared with the 2mg/kgQ3W group (*p* = 0.278), although these differences were not statistically significant (Table 3). The ORR did not differ significantly between the ≥4.6 mg/kg and <4.6 mg/kg groups (*p* = 0.607) (Table 4). However, it was significantly better in the Q < 3W group compared with the Q ≥ 3W group (*p* = 0.045). Across all regimens, approximately 50–60% of participants experienced tumor progression, and the DCR remained at 40–50%, regardless of nivolumab regimen, dosing interval, or dose. In a sub-analysis of participants who received nivolumab as first-line systemic therapy, the ORR showed similar trends: 240mgQ2W vs. 480mgQ4W (30.7% vs. 11.1%, respectively; *p* = 0.126); 2mg/kgQ3W vs. 3mg/kgQ2W (16.7% vs. 29.1%, respectively; *p* = 0.255); Q < 3W vs. Q ≥ 3W (29.3% vs. 14.8%, respectively; *p* = 0.065); and <4.6 mg/kg vs. ≥4.6 mg/kg (25.0% vs. 14.3%, respectively; *p* = 0.238).

In patients who were not continuing nivolumab treatment, reasons for discontinuing nivolumab treatment were compared by regimen (Table 5). In all nivolumab regimens, approximately 80% of patients discontinued treatment due to tumor progression or deterioration of their condition. There were no significant differences in reasons for discontinuation between regimens.

### 3.3. Progression-Free Survivals and Overall Survivals

**Figure 1:**
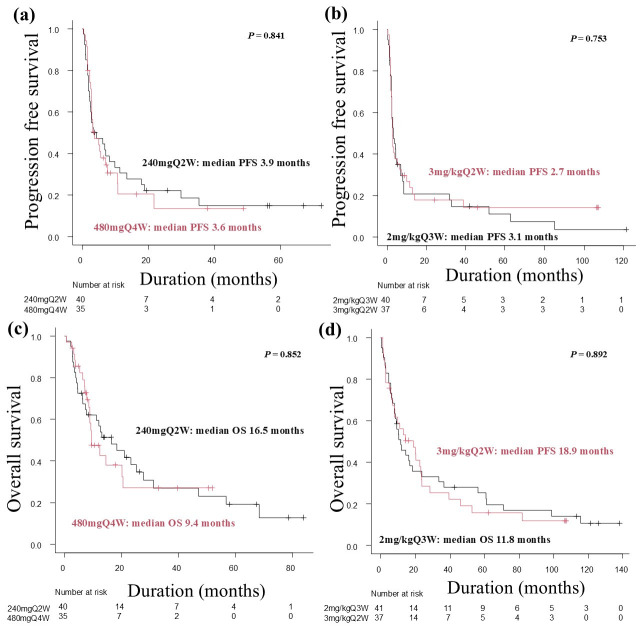
Kaplan–Meier analysis of progression-free survival (PFS) and overall survival (OS) by nivolumab regimen. (**a**) Kaplan–Meier analysis of PFS in the 240mgQ2W and 480mgQ4W groups. (**b**) Kaplan–Meier analysis of PFS in the 2mg/kgQ3W and 3mg/kgQ2W groups. (**c**) Kaplan–Meier analysis of OS in the 240mgQ2W and 480mgQ4W groups. (**d**) Kaplan–Meier analysis of OS in the 2mg/kgQ3W and 3mg/kgQ2W groups.

**Figure 2:**
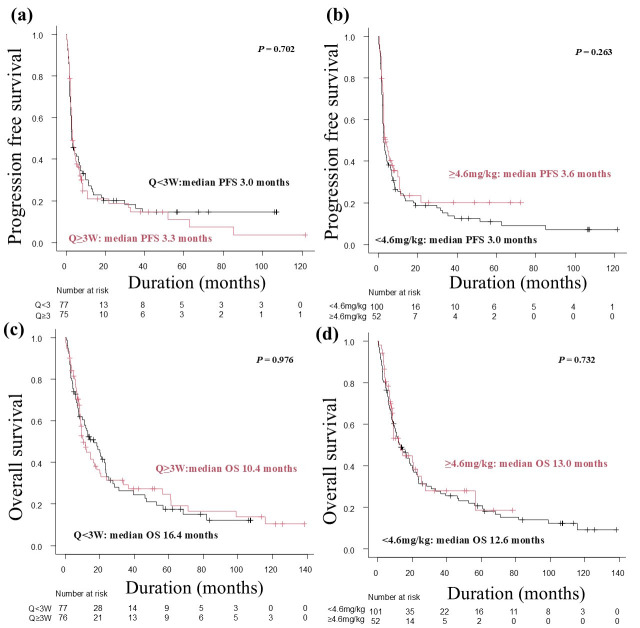
Kaplan–Meier analysis of progression-free survival (PFS) and overall survival (OS) by nivolumab dosing interval and dose per body weight. (**a**) Kaplan–Meier analysis of PFS in the Q < 3W and Q ≥ 3W groups. (**b**) Kaplan–Meier analysis of PFS in the <4.6 mg/kg and ≥4.6 mg/kg groups. (**c**) Kaplan–Meier analysis of OS in the Q < 3W and Q ≥ 3W groups. (**d**) Kaplan–Meier analysis of OS in the <4.6 mg/kg and ≥4.6 mg/kg groups.

No significant differences in PFS or OS were observed between the 240mgQ2W and 480mgQ4W groups (median PFS: 3.9 months vs. 3.6 months, respectively; log-rank test, *p* = 0.841; median OS: 16.5 months vs. 9.4 months, respectively; log-rank test, *p* = 0.852) (Figure 1a,c), or the 2mg/kgQ3W and 3mg/kgQ2W groups (median PFS: 3.1 months vs. 2.6 months, respectively; log-rank test, *p* = 0.753; median OS: 11.8 months vs. 18.9 months, respectively; log-rank test, *p* = 0.892) (Figure 1b,d). Similarly, no significant differences in PFS or OS were noted between the Q < 3W and Q ≥ 3W groups (median PFS: 3.0 months vs. 3.3 months, respectively; log-rank test, *p* = 0.702; median OS: 16.4 months vs. 10.4 months, respectively; log-rank test, *p* = 0.976) (Figure 2a,c), or the <4.6 mg/kg and ≥4.6 mg/kg groups (median PFS: 3.0 months vs. 3.6 months, respectively; log-rank test, *p* = 0.263; median OS: 12.6 months vs. 13.0 months, respectively; log-rank test, *p* = 0.732) (Figure 2b,d). Furthermore, there were no significant differences in the PFS rate at 6 and 12 months after the start of nivolumab treatment among each regimen group (Table 6).

### 3.4. Multivariate Analysis of Potential Prognostic Factors for Progression-Free Survival and Overall Survival

The multivariate analysis of potential prognostic factors for PFS and OS are shown in Table 7. The multivariate analysis showed that the primary site for MM (non-cutaneous, HR: 1.628, 95% confidence interval [CI]: 1.032–2.568, *p* = 0.036) and the treatment line of nivolumab (2nd and subsequent lines, HR: 2.388, 95% CI: 1.435–3.974, *p* < 0.001) were independent predictors of OS, whereas there were no independent predictors of PFS.

### 3.5. Toxicity

The incidence of any-grade irAEs was approximately 40–50% across all nivolumab regimens, dosing intervals, and dose categories, with no significant differences (Table 8 and Table 9). No significant differences were observed in the incidence of ≥3 grade irAEs regardless of regimen, interval, or dose. Regarding ≥3 grade irAEs, one participant (2.5%) each in the 240mgQ2W group experienced pneumonia, hepatitis, and bullous pemphigoid, and one participant (2.9%) in the 480mgQ4W group developed fever. In the 2mg/kgQ3W group, six participants (14.6%) developed pneumonia, one (2.4%) had bullous pemphigoid, and one (2.4%) had dermatitis. In the 3mg/kgQ2W group, one participant (2.7%) each developed hyperglycemia, pneumonia, hypopituitarism, and dermatitis. The median time to onset of ≥3 grade irAEs was 3.9 months (interquartile range [IQR]: 3.2–4.6) in the W group and 8.5 months (IQR: 1.6–8.9) in the Q ≥ 3W group (*p* = 0.874), and 5.1 months (IQR: 3.9–8.9) in the <4.6 mg/kg group and 2.1 months (IQR: 1.5–2.3) in the ≥4.6 mg/kg group (*p* = 0.069). Regarding the treatment of ≥3 grade irAEs, all patients except for those with endocrine disorders were administered systemic steroids, and no other immunosuppressants were administered. Two patients with pneumonia were administered steroid pulse therapy with 500 mg/day of methylprednisolone but were promptly switched to oral prednisolone. The other patients were treated with oral prednisolone from the beginning, with the median initial dose of oral prednisolone being 30.0 mg/day.

## 4. Discussion

This study evaluated the efficacy and side effects of four nivolumab regimens in Japanese participants with advanced MM. For patients with MM, nivolumab can be introduced via two regimens: 240mQ2W and 480mgQ4W. Understanding the efficacy and toxicity profiles of each regimen is essential for clinical decision making. The results demonstrate no significant difference in efficacy or toxicity between the two dosing strategies, supporting the appropriateness of either regimen for treatment.

Previous studies have examined nivolumab dosing intervals in patients with MM, particularly comparing weight-based regimens with fixed-dose regimens. Several reports [15,16,17] indicated that both regimens have similar efficacy, while one study suggested improved OS with fixed dosing [24]. Regarding toxicity, all studies consistently reported no significant differences in irAEs between groups [15,16,17,24]. Although this study did not directly compare weight-based and fixed-dose regimens, it found no significant differences in ORR, PFS, or OS.

Currently, fixed-dose regimens are favored due to their simplicity; however, real-world evidence on whether 240mgQ2W or the 480mgQ4W regimen should be used is limited.

Simeone et al. [15] reported that the 480mgQ4W regimen significantly prolonged OS compared with the 3mgQ2W and 240mgQ2W regimens in 124 patients with stage IV MM in Italy (HR: 0.48, 95% CI: 0.24–0.96, *p* = 0.04). Their study was the first real-world comparison of these regimens; this study is the second. In contrast to their findings, our results showed no difference in PFS or OS between the two regimens. Although such subgroup considerations are rarely addressed in other cancers, Murashima et al. [25] compared 240mgQ2W with 480mgQ4W in advanced esophageal squamous cell carcinoma and found no differences in ORR, PFS, OS, or incidence of irAEs. Together with the present findings, this suggests that differences between regimens may be less pronounced in the Japanese population. It has been reported that the response rate to ICIs varies by race, and East Asians are often reported to benefit less from ICI treatment than Caucasians [12,13,14]. In a comparison of response rates to nivolumab monotherapy, in stage IV MM, the response rate in Japanese patients is about 15–30% [12,14], while in Westerners, it has been reported to be over 40% [3,4]. In addition to differences in race, differences in subtype also have a significant impact on the efficacy of ICIs. In a study reporting on 7442 Japanese patients with MM [26], 40.7% had acral MM and 9.5% had mucosal MM, accounting for half of the total. However, it has been reported that in Caucasians, acral MM is about 1% [27] and mucosal MM is about 1–3% [28,29], and the proportion of MM subtypes by race varies greatly. The response rate to nivolumab monotherapy is about 20% for mucosal MM [30] and about 30% for acral MM [31], which is known to be lower than the ORR of MM, which is over 40% [3,5]. More than half of the patients in this study had non-cutaneous MM (including acral MM), which may have reduced the response rate of all nivolumab regimens and reduced the difference in efficacy between the nivolumab regimens.

In addition, approximately 30% of the total patients in this study used nivolumab as a second-line or subsequent lines of therapy. It has been reported that the response rate of ICIs is lower in second-line or subsequent treatments compared with first line [32], and in this study, the treatment line of nivolumab was also an independent predictor of OS (Table 7). Since there was no difference in prior systemic therapy between each regimen, the possibility of bias due to prior treatment is low. However, as with non-cutaneous MM, it may have significantly reduced the response rate of all nivolumab regimens overall. Further MM subgroup and race-specific analyses are warranted.

With respect to toxicity, several studies have evaluated the 240mgQ2W and 480mgQ4W regimens in MM. Truong et al. [18] reported on the toxicity of nivolumab administered every 2 weeks (maximum dose, 240 mg/body) and every 4 weeks (maximum dose, 480 mg/body) in 71 participants with adjuvant or metastatic MM and found no significant differences in incidence between regimens, although irAEs tended to occur later in the Q4W group. Similarly, this study observed a delayed onset of severe irAEs in the Q4W group, though the difference was not significant (*p* = 0.874). Samlowski et al. [20] also reported no significant differences in irAEs across 240mgQ2W, 480mgQ4W, and 3mg/kgQ2W regimens in 191 participants with MM undergoing adjuvant therapy. In a broader analysis involving solid tumors, Elijah et al. [33] found no overall differences in irAE incidence among those taking 240mgQ2W and 480mgQ4W and those who switched regimens; however, they reported fewer severe irAEs in the 480mgQ4W group. Colitis was more frequent in the 240mgQ2W group, while pruritus was more common in the 480mgQ4W group, indicating that regimen-specific adverse effects may exist. Since toxicity profiles can vary by carcinoma type [34,35], further studies focusing specifically on MM are needed. In this study, irAEs of any grade occurred in approximately 40–50% of patients, and severe irAEs in about 10%, with no significant differences between regimens. The lower overall irAE rate compared with prospective studies [3,4,5] may be attributable to the retrospective nature of this study, where minor irAEs may not have been captured. This study suggests that there is little need to consider differences in side effects when selecting a nivolumab regimen for patients with MM.

A notable feature of this study was the additional comparison between dosing intervals (Q ≥ 3W vs. Q < 3W) and dosage groups (≥4.6 mg/kg vs. <4.6 mg/kg) to separately evaluate the effects of administration frequency and dose. These results suggest that the incidence of irAEs is not dependent on either factor. However, shorter administration intervals may be associated with improved short-term efficacy, although no long-term advantage was observed. One reason for the lack of significant differences in PFS and OS despite the better response rates in the shorter interval groups (Q < 3, 240mgQ2W, and 3mg/kgQ2W) may be that there were no significant differences in DCR. Eftychia et al. [36] reported a significantly better OS in 265 patients with unresectable stage IV MM treated with ICIs in the order of CR, PR, SD, and progressive disease (*p* < 0.001 for each). However, Kaplan–Meier curves showed no significant difference between the PR and SD groups in the first few years of treatment. In terms of PFS, there was a significant difference between the SD and progressive disease groups (*p* < 0.001), but no significant difference between the PR and SD groups (*p* = 0.066). This fact suggests that DCR may be more strongly associated with long-term prognosis than ORR. Considering both our findings and prior research, the nivolumab regimen selection for Japanese patients with MM should prioritize individual factors such as hospital visit frequency and treatment cost rather than differences in efficacy or side effects. However, if the patient has symptoms associated with the primary or metastatic lesions and symptoms can be expected to improve with tumor shrinkage, a short-interval nivolumab regimen may be preferable.

This study is the first to compare the efficacy of the 240mgQ2W and 480mgQ4W regimens in Asian patients with MM. Our results suggest that the 240mgQ2W regimen may be preferable for patients who benefit from short-term tumor shrinkage, while neither regimen is significantly different in terms of long-term response and side effects, and the choice of regimen should be based on patient preference. The findings contribute valuable real-world data to inform treatment decisions, especially in East Asia. Nonetheless, this study has several limitations. As a retrospective cohort study, it is subject to selection bias and the potential omission of minor irAEs. Additionally, the single-center design and small sample size may limit its generalizability and statistical power, particularly for inter-regimen comparisons. In addition, there was a large bias in sample size when comparing the ≥4.6 mg/kg group with the 4.6 mg/kg group, which may have also hindered accurate statistical analysis. To address these issues, further validation with a larger sample size and/or prospective studies may be required.

## 5. Conclusions

Shorter dosing intervals of nivolumab may yield short-term benefits in Japanese patients with advanced MM. However, no significant differences in long-term efficacy or toxicity were observed between regimens. Hence, for patients with advanced MM in East Asia, both 240mgQ2W and 480mgQ4W regimens are viable options. Further studies stratified by race and melanoma subtype are warranted.

## Figures and Tables

**Table 1:** Baseline characteristics of patients with malignant melanoma grouped by nivolumab regimen.

	Patient Group (%)		*p*-Value	Patient Group (%)		*p*-Value
Characteristic	240mgQ2W	480mgQ4W		2mg/kgQ3W	3mg/kgQ2W	
Patients, *n* (%)	40 (53.3)	35 (46.7)		41 (52.6)	37 (47.4)	0.908
Age, years, Median [range]	69.5 [34.0, 92.0]	67.0 [34.0, 84.0]	0.718	66.0 [28.0, 88.0]	66.0 [27.0, 87.0]	
Sex			0.819			0.656
Male	21 (52.5)	17 (48.6)		21 (51.2)	21 (56.8)	
Female	19 (47.5)	18 (51.4)		20 (48.8)	16 (43.2)	
ECOG-PS			0.364			0.553
0–1	36 (90.0)	34 (97.1)		33 (80.5)	32 (86.5)	
≥2	4 (10.0)	1 (2.9)		8 (19.5)	5 (13.5)	
Primary site			0.853			0.459
Cutaneous	16 (40.0)	11 (31.4)		19 (46.3)	11 (29.7)	
Acral	8 (20.0)	6 (17.1)		4 (9.8)	8 (21.6)	
Mucosal	13 (32.5)	13 (37.1)		13 (31.7)	14 (37.8)	
Uveal	2 (5.0)	3 (8.6)		2 (4.9)	1 (2.7)	
Unknown	1 (2.5)	2 (5.7)		3 (7.3)	3 (8.1)	
Details of stage Ⅳ			0.542			0.622
M1a	7 (17.5)	7 (20.0)		6 (14.6)	6 (16.2)	
M1b	9 (22.5)	4 (11.4)		4 (9.8)	7 (18.9)	
M1c	21 (52.5)	19 (54.3)		27 (65.9)	22 (59.5)	
M1d	3 (7.5)	5 (14.3)		4 (9.8)	2 (5.4)	
LDH value			0.252			
<ULN	24 (60.0)	16 (45.7)		18 (43.9)	17 (45.9)	
≥ULN	16 (40.0)	19 (54.3)		23 (56.1)	20 (54.1)	
BRAF			0.158			**0.031** *
Mutant	3 (7.5)	8 (22.9)		7 (17.1)	5 (13.5)	
Wild	31 (77.5)	24 (68.6)		16 (39.0)	25 (67.6)	
Not investigated	6 (15.0)	3 (8.6)		18 (43.9)	7 (18.9)	
Number of organs involved			0.795			0.191
1	21 (52.5)	16 (45.7)		16 (39.0)	20 (54.1)	
2	9 (22.5)	10 (28.6)		14 (34.1)	6 (16.2)	
≥3	10 (25.0)	9 (25.7)		11 (26.8)	11 (29.7)	
Surgery for primary site			0.611			0.812
Yes	30 (75.0)	24 (68.6)		28 (68.3)	24 (64.9)	
Adjuvant therapy			1.000			0.066
Yes	10 (25.0)	8 (22.9)		13 (31.7)	5 (13.5)	
Number of previous treatment lines (for metastatic/unresectable)			0.476			0.882
0	26 (65.0)	18 (51.4)		36 (87.8)	32 (86.5)	
1	11 (27.5)	13 (37.1)		4 (9.8)	3 (8.1)	
≥2	3 (7.5)	4 (11.4)		1 (2.4)	2 (5.4)	
Previous systematic therapy						
BRAF/MEK inhibitor	1 (2.5)	5 (14.3)	0.092	0 (0)	1 (2.7)	0.474
Immune-checkpoint inhibitor	14 (35.0)	15 (42.9)	0.635	3 (7.3)	4 (10.8)	0.702
Cytotoxic anticancer drugs	0 (0)	1 (2.9)	0.467	3 (7.3)	1 (2.7)	0.617
Median follow-up time (months) [IQR]	12.8 [4.5–25.5]	8.8 [5.8–13.4]	0.276	10.4 [5.9–42.4]	14.2 [5.1–23.8]	0.837

BRAF/MEK, B-rapidly accelerated fibrosarcoma and mitogen-activated protein kinase; ECOG-PS, Eastern Cooperative Oncology Group Performance Status; IQR, interquartile range; LDH, lactate dehydrogenase; ULN, upper limit of normal. * Bold letters indicate statistically significant differences: *p* < 0.05.

**Table 2:** Baseline characteristics of patients with malignant melanoma classified by nivolumab dosing interval and dose per body weight.

	Patient Group (%)		*p*-Value	Patient Group (%)		*p*-Value
Characteristic	Q < 3W	Q ≥ 3W		<4.6mg/kg	≥4.6mg/kg	
Patients, *n* (%)	77 (50.3)	76 (49.7)		101 (66.0)	52 (34.0)	0.304
Age, years, Median [range]	68.0 [27.0, 92.0]	67.0 [28.0, 88.0]	0.693	68.0 [27.0, 88.0]	68.0 [34.0, 92.0]	
Sex			0.629			0.174
Male	42 (54.5)	38 (50.0)		57 (56.4)	23 (44.2)	
Female	35 (45.5)	38 (50.0)		44 (43.6)	29 (55.8)	
ECOG-PS			1.000			0.118
0–1	68 (88.3)	67 (88.2)		86 (85.1)	49 (94.2)	
≥2	9 (11.7)	9 (11.8)		15 (14.9)	3 (5.8)	
Primary site			0.717			0.711
Cutaneous	27 (35.1)	30 (39.5)		40 (39.6)	17 (32.7)	
Acral	16 (20.8)	10 (13.2)		17 (16.8)	9 (17.3)	
Mucosal	27 (35.1)	26 (34.2)		33 (32.7)	20 (38.5)	
Uveal	3 (3.9)	5 (6.6)		4 (4.0)	4 (7.7)	
Unknown	4 (5.2)	5 (6.6)		7 (6.9)	2 (3.8)	
Details of stage Ⅳ			0.275			0.285
M1a	13 (16.9)	13 (16.9)		17 (16.8)	9 (17.3)	
M1b	16 (20.8)	8 (10.5)		16 (15.8)	8 (15.4)	
M1c	43 (55.8)	46 (60.5)		62 (61.4)	27 (51.9)	
M1d	5 (6.5)	9 (11.8)		6 (5.9)	8 (15.4)	
LDH value			0.333			0.614
<ULN	41 (53.2)	34 (44.7)		48 (47.5)	27 (51.9)	
≥ULN	36 (46.8)	42 (55.3)		53 (52.5)	25 (48.1)	
BRAF			0.036 *			0.354
Mutant	8 (10.4)	15 (19.7)		14 (13.9)	9 (17.3)	
Wild	56 (72.7)	40 (52.6)		61 (60.4)	35 (67.3)	
Not investigated	13 (16.9)	21 (27.6)		26 (25.7)	8 (15.4)	
Number of organs involved			0.202			0.837
1	41 (53.2)	32 (42.1)		50 (49.5)	23 (44.2)	
2	15 (19.5)	24 (31.6)		25 (24.8)	14 (26.9)	
≥3	21 (27.3)	20 (26.3)		26 (25.7)	15 (28.8)	
Surgery for primary site			0.862			0.853
Yes	54 (70.1)	52 (68.4)		69 (68.3)	37 (71.2)	
Adjuvant therapy			0.258			0.547
Yes	15 (19.5)	21 (27.6)		22 (21.8)	14 (26.9)	
Number of previous treatment lines (for metastatic/unresectable)			0.851			**<0.001** *
0	58 (75.3)	54 (71.1)		84 (83.2)	28 (53.8)	
1	14 (18.2)	17 (22.4)		11 (10.9)	20 (38.5)	
≥2	5 (6.5)	5 (6.6)		6 (5.9)	4 (7.7)	
Previous systematic therapy						
BRAF/MEK inhibitor	2 (2.6)	5 (6.6)	0.276	2 (2.0)	5 (9.6)	**0.045**
Immune-checkpoint inhibitor	18 (23.4)	18 (23.7)	1.000	14 (13.9)	22 (42.3)	**<0.001**
Cytotoxic anticancer drugs	1 (1.3)	4 (5.3)	0.209	4 (4.0)	1 (1.9)	0.662
Median follow-up time (months) [IQR]	13.1 [5.0–25.2]	9.3 [5.9–21.3]	0.602	12.6 [5.6–31.2]	8.8 [5.2–20.3]	0.252

BRAF/MEK, B-rapidly accelerated fibrosarcoma and mitogen-activated protein kinase; ECOG-PS, Eastern Cooperative Oncology Group Performance Status; IQR, interquartile range; LDH, lactate dehydrogenase; ULN, upper limit of normal. * Bold letters indicate statistically significant differences: *p* < 0.05.

**Table 3:** Objective response rates by nivolumab regimen.

	Patient Group (%)		*p*-Value	Patient Group (%)		*p*-Value
	240mgQ2W *n* = 40	480mgQ4W *n* = 35		2mg/kgQ3W *n* = 41	3mg/kgQ2W *n* = 37	
Best overall response			0.095			0.228
Complete response	1 (2.5)	2 (5.7)		0 (0.0)	2 (5.4)	
Partial response	10 (25.0)	2 (5.7)		6 (14.6)	7 (18.9)	
Stable response	8 (20.0)	11 (31.4)		13 (31.7)	6 (16.2)	
Progressive disease	21 (52.5)	20 (57.1)		22 (53.7)	22 (59.5)	
Objective response rate	27.5%	11.4%	0.082	14.6%	24.3%	0.278
Disease control rate	47.5%	42.8%	0.687	46.3%	40.5%	0.606

**Table 4:** Objective response rates by nivolumab dosing interval and dose per body weight.

	Patient Group (%)		*p*-Value	Patient Group (%)		*p*-Value
	Q < 3W *n* = 77	Q ≥ 3W *n* = 76		<4.6mg/kg *n*= 101	≥4.6mg/kg *n* = 52	
Best overall response			0.103			0.446
Complete response	3 (3.9)	2 (2.6)		2 (2.0)	3 (5.8)	
Partial response	17 (22.1)	8 (10.5)		19 (18.8)	6 (11.5)	
Stable response	14 (18.2)	24 (31.6)		24 (23.8)	14 (26.9)	
Progressive disease	43 (55.8)	42 (55.3)		56 (55.4)	29 (55.8)	
Objective response rate	26.0%	13.1%	0.045 *	20.8%	17.3%	0.607
Disease control rate	44.2%	44.7%	0.910	44.6%	44.2%	0.970

* Bold letters indicate statistically significant differences: *p* < 0.05.

**Table 5:** Reasons for terminating nivolumab treatment by regimen in patients with malignant melanoma.

	Patient Group (%)		*p*-Value	Patient Group (%)		*p*-Value
	240mgQ2W *n* = 37	480mgQ4W *n* = 31		2mg/kgQ3W *n* = 41	3mg/kgQ2W *n* = 36	
Reason for discontinuing nivolumab			0.663			0.108
Complete response	1 (2.7)	1 (3.2)		0 (0.0)	1 (2.8)	
Immune-related adverse events	5 (13.5)	2 (6.5)		8 (19.5)	4 (11.1)	
Progressive disease or patient deterioration	31 (83.8)	27 (87.1)		33 (80.5)	28 (77.8)	
Patient decision	0 (0.0)	1 (3.2)		0 (0.0)	3 (8.3)	

**Table 6:** Progression-free survival rates at 6 and 12 months after the start of nivolumab treatment for each nivolumab regimen.

	Patient Group (%)		*p*-Value	Patient Group (%)		*p*-Value
	240mgQ2W *n* = 40	480mgQ4W *n* = 35		2mg/kgQ3W *n* = 41	3mg/kgQ2W *n* = 37	
6-month PFS rate	17 (42.5)	12 (34.3)	0.487	13 (31.7)	13 (35.1)	0.812
12-month PFS rate	11 (27.5)	4 (11.4)	0.147	7 (17.1)	8 (21.6)	0.775

PFS, progression-free survival.

**Table 7:** Multivariate analysis of potential prognostic factors for progression-free survival and overall survival.

	PFS			OS		
	Hazard Ratio	95% CI	*p*-Value *	Hazard Ratio	95% CI	*p*-Value *
Age	1.009	0.994–1.025	0.249	1.013	0.996–1.030	0.145
Sex						
Female	Reference			Reference		
Male	1.168	0.806–1.693	0.412	1.350	0.912–1.999	0.133
Primary site						
Cutaneous	Reference			Reference		
Non-cutaneous	1.283	0.818–2.014	0.278	1.628	1.032–2.568	**0.036**
BRAF						
Wild	Reference			Reference		
Mutant	1.002	0.528–1.901	0.996	0.619	0.325–1.179	0.145
Not investigated	0.828	0.511–1.343	0.445	1.060	0.656–1.711	0.813
Nivolumab regimens						
240mgQ2W	Reference			Reference		
480mgQ4W	0.959	0.561–1.642	0.881	1.028	0.572–1.847	0.928
2mg/kgQ3W	1.460	0.848–2.513	0.172	1.316	0.758–2.287	0.329
3mg/kgQ2W	1.282	0.761–2.160	0.350	1.299	0.759–2.222.	0.3294
Nivolumab treatment line						
1st line	Reference			Reference		
2nd and subsequent lines	1.647	0.975–2.782	0.062	2.388	1.435–3.974	**<0.001**

BRAF, B-rapidly accelerated fibrosarcoma; CI, confidence interval; OS, overall survival; PFS, progression-free survival. * Bold letters indicate statistically significant differences: *p* < 0.05.

**Table 8:** Immune-related adverse events by nivolumab regimen.

	Patient Group (%)		*p*-Value	Patient Group (%)		*p*-Value
	240mgQ2W *n* = 40	480mgQ4W *n* = 35		2mg/kgQ3W *n* = 41	240mgQ2W *n* = 40	
Any-grade immune-related adverse events			0.819			1.000
Yes	17 (42.5)	16 (45.7)		19 (46.3)	18 (48.6)	
No	23 (57.5)	19 (54.3)		22 (53.7)	19 (51.4)	
≥3 grade immune-related adverse events			0.618			0.356
Yes	3 (7.5)	1 (2.9)		8 (19.5)	4 (10.8)	
No	37 (92.5)	34 (97.1)		33 (80.5)	33 (89.2)	

**Table 9:** Immune-related adverse events by nivolumab dosing interval and dose per body weight.

	Patient Group (%)		*p*-Value	Patient Group (%)		*p*-Value
	Q < 3W *n* = 77	Q ≥ 3W *n* = 76		<4.6mg/kg *n* = 101	≥4.6mg/kg *n* = 52	
Any-grade immune-related adverse events			1.000			0.864
Yes	35 (45.5)	35 (46.1)		47 (46.5)	23 (44.2)	
No	42 (54.5)	41 (53.9)		54 (53.5)	29 (55.8)	
≥3 grade immune-related adverse events			0.608			0.265
Yes	7 (9.1)	9 (11.8)		13 (12.9)	3 (5.8)	
No	70 (90.9)	67 (88.2)		88 (87.1)	49 (94.2)	

## Data Availability

The raw data supporting the conclusions of this article are available from the authors upon request.

## Abbreviations

The following abbreviations are used in this manuscript:

BRAF/MEK	B-rapidly accelerated fibrosarcoma and mitogen-activated protein kinase
CR	Complete response
ICI	Immune-checkpoint inhibitor
irAE	Immune-related adverse event
ORR	Objective response rate
OS	Overall survival
PFS	Progression-free survival
PR	Partial response
SD	Stable disease

## References

[1] Cybulska-Stopa B., Piejko K., Pacholczak R., Domagała-Haduch M., Drosik-Kwaśniewska A., Rolski J., Wiktor-Mucha P., Zemełka T. (2020). Real-world treatment practice in patients with advanced melanoma. Contemp. Oncol..

[2] Malmberg R., Zietse M., Dumoulin D.W., Hendrikx J.J.M.A., Aerts J.G.J.V., van der Veldt A.A.M., Koch B.C.P., Sleijfer S., van Leeuwen R.W.F. (2022). Alternative dosing strategies for immune checkpoint inhibitors to improve cost-effectiveness: A special focus on nivolumab and pembrolizumab. Lancet Oncol..

[3] Robert C., Long G.V., Brady B., Dutriaux C., Maio M., Mortier L., Hassel J.C., Rutkowski P., McNeil C., Kalinka-Warzocha E. (2015). Nivolumab in previously untreated melanoma without BRAF mutation. N. Engl. J. Med..

[4] Wolchok J.D., Kluger H., Callahan M.K., Postow M.A., Rizvi N.A., Lesokhin A.M., Segal N.H., Ariyan C.E., Gordon R.-A., Reed K. (2013). Nivolumab plus ipilimumab in advanced melanoma. N. Engl. J. Med..

[5] Larkin J., Chiarion-Sileni V., Gonzalez R., Grob J.J., Cowey C.L., Lao C.D., Schadendorf D., Dummer R., Smylie M., Rutkowski P. (2015). Combined nivolumab and ipilimumab or monotherapy in untreated melanoma. N. Engl. J. Med..

[6] de Miguel M., Calvo E. (2020). Clinical Challenges of Immune Checkpoint Inhibitors. Cancer Cell.

[7] Yamazaki N., Kiyohara Y., Uhara H., Uehara J., Fujimoto M., Takenouchi T., Otsuka M., Uchi H., Ihn H., Minami H. (2017). Efficacy and safety of nivolumab in Japanese patients with previously untreated advanced melanoma: A phase II study. Cancer Sci..

[8] Long G.V., Atkinson V., Lo S., Sandhu S., Guminski A.D., Brown M.P., Wilmott J.S., Edwards J., Gonzalez M., Scolyer R.A. (2018). Combination nivolumab and ipilimumab or nivolumab alone in melanoma brain metastases: A multicentre randomised phase 2 study. Lancet Oncol..

[9] Weber J., Mandala M., Del Vecchio M., Gogas H.J., Arance A.M., Cowey C.L., Dalle S., Schenker M., Chiarion-Sileni V., Marquez-Rodas I. (2017). Adjuvant nivolumab versus ipilimumab in resected stage III or IV melanoma. N. Engl. J. Med..

[10] Pacholczak-Madej R., Grela-Wojewoda A., Puskulluoglu M., Lompart J., Las-Jankowska M., Krawczak K., Wrona E., Zaręba L., Żubrowska J., Walocha J. (2022). Early Effects of Nivolumab and Ipilimumab Combined Immunotherapy in the Treatment of Metastatic Melanoma in Poland: A Multicenter Experience. Biomedicines.

[11] Sznol M., Ferrucci P.F., Hogg D., Atkins M.B., Wolter P., Guidoboni M., Lebbé C., Kirkwood J.M., Schachter J., Daniels G.A. (2017). Pooled analysis safety profile of nivolumab and ipilimumab combination therapy in patients with advanced melanoma. J. Clin. Oncol..

[12] Yamazaki N., Takenouchi T., Nakamura Y., Takahashi A., Namikawa K., Kitano S., Fujita T., Kubota K., Yamanaka T., Kawakami Y. (2021). Prospective observational study of the efficacy of nivolumab in Japanese patients with advanced melanoma (CREATIVE study). Jpn. J. Clin. Oncol..

[13] Mao L., Qi Z., Zhang L., Guo J., Si L. (2021). Immunotherapy in acral and mucosal melanoma: Current status and future directions. Front. Immunol..

[14] Namikawa K., Ito T., Yoshikawa S., Yoshino K., Kiniwa Y., Ohe S., Isei T., Takenouchi T., Kato H., Mizuhashi S. (2023). Systemic therapy for Asian patients with advanced BRAF V600-mutant melanoma in a real-world setting: A multi-center retrospective study in Japan (B-CHECK-RWD study). Cancer Med..

[15] Bei D., Osawa M., Uemura S., Ohno T., Gobburu J., Roy A., Hasegawa M. (2020). Benefit-risk assessment of nivolumab 240 mg flat dose relative to 3 mg/kg Q2W regimen in Japanese patients with advanced cancers. Cancer Sci..

[16] Staender H.F., Langan E.A. (2025). Fixed-dose versus weight-adapted immune checkpoint inhibitor therapy in melanoma: A retrospective monocentric analysis of efficacy and immune-related adverse events. Cancers.

[17] Campo Le Brun I., Dalle S., Mortier L., Dereure O., Dalac Rat S., Dutriaux C., Leccia M.T., Legoupil D., Montaudié H., De Quatrebarbes J. (2025). Methods of nivolumab administration in advanced melanoma: A comparison of patients’ clinical outcomes treated with flat dose or weight-adjusted dose, a multicenter observational study. Cancer.

[18] Truong J., Yeung S.S.T., Kletas V., de Lemos M., Schaff K., Nakashima L. (2024). Utilization and toxicity patterns of 2-weekly (Q2W) versus 4-weekly (Q4W) nivolumab for treatment of adjuvant and metastatic melanoma at BC Cancer. J. Oncol. Pharm. Pract..

[19] Samlowski W., Robert N.J., Chen L., Schenkel B., Davis C., Moshyk A., Kotapati S., Poretta T., Weber J.S. (2023). Real-world nivolumab dosing patterns and safety outcomes in patients receiving adjuvant therapy for melanoma. Cancer Med..

[20] Simeone E., Mallardo D., Giannarelli D., Festino L., Vanella V., Trojaniello C., Vitale M.G., Palla M., Scarpato L., Capone M. (2020). Correlation of nivolumab 480 mg Q4W with better survival than other nivolumab monotherapy schedule in metastatic melanoma patients. J. Clin. Oncol..

[21] Gershenwald J.E., Scolyer R.A., Hess K.R., Sondak V.K., Long G.V., Ross M.I., Lazar A.J., Faries M.B., Kirkwood J.M., McArthur G.A. (2017). Melanoma staging: Evidence-based changes in the American Joint Committee on Cancer eighth edition cancer staging manual. CA Cancer J. Clin..

[22] Eisenhauer E.A., Therasse P., Bogaerts J., Schwartz L.H., Sargent D., Ford R., Dancey J., Arbuck S., Gwyther S., Mooney M. (2009). New response evaluation criteria in solid tumours: Revised RECIST guideline (version 1.1). Eur. J. Cancer.

[23] Shah S. (2022). Common Terminology Criteria for Adverse Events.

[24] Leroy M., Desmedt E., Deramoudt L., Vasseur M., Odou P., Béhal H., Décaudin B., Mortier L., Simon N. (2024). Retrospective comparison of a weight-based dose every 2 weeks with a fixed dose every month: A real-life analysis of nivolumab in the treatment of advanced melanoma. Melanoma Res..

[25] Murashima Y., Yamamoto S., Hirose T., Kadono T., Ikeda G., Ohara A., Itoyama M., Yokoyama K., Honma Y., Ishiyama K. (2024). Efficacy and safety of salvage-line nivolumab monotherapy for advanced esophageal squamous cell carcinoma: Comparison of 240 mg versus 480 mg doses. J. Gastrointest. Cancer.

[26] Fujisawa Y., Yoshikawa S., Takenouchi T., Mori S., Asai J., Uhara H., Ichigosaki Y., Fujimura T., Nakamura Y., Nakamura Y. (2025). Melanoma skin cancer statistics derived from 7442 Japanese patients: Japanese melanoma study. Int. J. Clin. Oncol..

[27] Huang K., Fan J., Misra S. (2020). Acral Lentiginous Melanoma: Incidence and Survival in the United States, 2006–2015, an Analysis of the SEER Registry. J. Surg. Res..

[28] Sergi M.C., Filoni E., Triggiano G., Cazzato G., Internò V., Porta C., Tucci M. (2023). Mucosal Melanoma: Epidemiology, Clinical Features, and Treatment. Curr. Oncol. Rep..

[29] Boer F.L., Ho V.K.Y., Louwman M.W.J., Schrader A.M.R., Zuur C.L., Blank C.U., van Poelgeest M.I.E., Kapiteijn E.H.W. (2023). Trends in Incidence and Survival of 1496 Patients with Mucosal Melanoma in The Netherlands (1990–2019). Cancers.

[30] D’Angelo S.P., Larkin J., Sosman J.A., Lebbé C., Brady B., Neyns B., Schmidt H., Hassel J.C., Hodi F.S., Lorigan P. (2017). Efficacy and Safety of Nivolumab Alone or in Combination with Ipilimumab in Patients with Mucosal Melanoma: A Pooled Analysis. J. Clin. Oncol..

[31] Nathan P., Ascierto P.A., Haanen J., Espinosa E., Demidov L., Garbe C., Guida M., Lorigan P., Chiarion-Sileni V., Gogas H. (2019). Safety and efficacy of nivolumab in patients with rare melanoma subtypes who progressed on or after ipilimumab treatment: A single-arm, open-label, phase II study (CheckMate 172). Eur. J. Cancer.

[32] Ascierto P.A., Mandalà M., Ferrucci P.F., Guidoboni M., Rutkowski P., Ferraresi V., Arance A., Guida M., Maiello E., Gogas H. (2023). Sequencing of Ipilimumab Plus Nivolumab and Encorafenib Plus Binimetinib for Untreated *BRAF*-Mutated Metastatic Melanoma (SECOMBIT): A Randomized, Three-Arm, Open-Label Phase II Trial. J. Clin. Oncol..

[33] Elijah J., Puzanov I., Cresanti B., Hamad L., Attwood K., Catalfamo K., Riebandt G. (2024). Evaluation of safety outcomes between nivolumab regimens with differing dosing patterns. J. Oncol. Pharm. Pract..

[34] Khoja L., Day D., Chen T.W.W., Siu L.L., Hansen A.R. (2017). Tumour- and class-specific patterns of immune-related adverse events of immune checkpoint inhibitors: A systematic review. Ann. Oncol..

[35] Xu Q., Hu J., Wang Y., Wang Z. (2024). The role of tumor types in immune-related adverse events. Clinical and Translational Oncology.

[36] Chatziioannou E., Leiter U., Thomas I., Keim U., Seeber O., Meiwes A., Boessenecker I., Gonzalez S.S., Torres F.M., Niessner H. (2023). Features and Long-Term Outcomes of Stage IV Melanoma Patients Achieving Complete Response Under Anti-PD-1-Based Immunotherapy. Am. J. Clin. Dermatol..

